# Higher Phytohormone Contents and Weaker Phytohormone Signal Transduction Were Observed in Cold-Tolerant Cucumber

**DOI:** 10.3390/plants11070961

**Published:** 2022-04-01

**Authors:** Radwa Salah, Rui-Jin Zhang, Shi-Wei Xia, Shan-Shan Song, Qian Hao, Mustafa H. Hashem, Huan-Xiu Li, Yu Li, Xi-Xiang Li, Yun-Song Lai

**Affiliations:** 1 College of Horticulture, Sichuan Agricultural University, Chengdu 611130, China; sradwa461@gmail.com (R.S.); 1551490472@163.com (R.-J.Z.); xia-sw@stu.sicau.edu.cn (S.-W.X.); songss@stu.sicau.edu.cn (S.-S.S.); 2020205020@stu.sicau.edu.cn (Q.H.); Darsh30@hotmail.Com (M.H.H.); 10650@sicau.edu.cn (H.-X.L.); liyu@sicau.edu.cn (Y.L.); 2 Faculty of Agriculture, Minya University, Minya 61511, Egypt; 3 Central Lab. of Organic Agriculture, Agricultural Research Center, Giza 12619, Egypt; 4 Institution of Vegetables and Flowers, Chinese Academy of Agricultural Sciences, South Zhongguancun Street 12, Beijing 100081, China; lixixiang@caas.cn

**Keywords:** *Cucumis sativus*, RNA-seq, transcriptome, cold tolerance, plant hormone

## Abstract

Cucumbers (*Cucumis sativus* L.) originated from the South Asian subcontinent, and most of them are fragile to cold stress. In this study, we evaluated the cold tolerance of 115 cucumber accessions and screened out 10 accessions showing high resistance to cold stress. We measured and compared plant hormone contents between cold-tolerant cucumber CT90R and cold-sensitive cucumber CT57S in cold treatment. Most of the detected plant hormones showed significantly higher content in CT90R. To elucidate the role of plant hormones, we compared the leaf- and root-transcriptomes of CT90R with those of CT57S in cold stress treatment. In leaves, there were 1209 differentially expressed genes (DEGs) between CT90R and CT57S, while there were 703 in roots. These DEGs were not evenly distributed across the chromosomes and there were significant enrichments at particular positions, including *qLTT6.2*, a known QTL controlling cucumber cold tolerance. The GO and KEGG enrichment analysis showed that there was a significant difference in the pathway of plant hormone transductions between CT90R and CT57S in leaves. In short, genes involved in plant hormone transductions showed lower transcription levels in CT90R. In roots, the most significantly different pathway was phenylpropanoid biosynthesis. CT90R seemed to actively accumulate more monolignols by upregulating *cinnamyl-alcohol* *dehydrogenase* (*CAD*) genes. These results above suggest a new perspective on the regulation mechanism of cold tolerance in cucumbers.

## 1. Introduction

Low temperature is one of the major environmental factors limiting agricultural productivity and the geographic distribution of plant species [1]. Cucumbers (*Cucumis sativus* L.) trace their genealogy back to tropical regions and the ideal temperature for growing is 20–35 °C. Temperatures below 16 °C and 8 °C result in growth suppression and chilling injury, respectively [2,3]. In China, an unusually cold spell in an otherwise warm early spring badly can affect cucumber seedlings and lead to a decline in fruit yield and quality. Lower temperature is also known to widely increase the female flower ratio in cucumber germplasm, which results in a higher fruit yield in spring than in early autumn [4]. The exploration and utilization of cold-tolerant cucumber germplasm is fundamental in cucumber breeding.

The interaction of cold stress and plants has been widely and thoroughly studied for a long history; cold stress response and cold acclimation are the two hot topics [5,6]. Presentative damage caused by cold include suppressed photosynthesis, decreased cell membrane fluidity, and increased oxidant compounds. Significant efforts were also made to determine genes and proteins regulated by cold stress, and profile their response to cold stress [7,8,9]. Gene modification of *cold-regulated* (*COR*) genes can change cold resistance in plants, as demonstrated in *Arabidopsis* [10]. Plant hormones play critical roles in cold stress and plant interactions; abscisic acid (ABA) is usually taken as a central plant hormone in cold stress studies [5,9]. The application of ABA and CaCl_2_ can significantly enhance cold tolerance in cucumbers [11]. Many other studies reported the cross talk of signal transduction between ABA and other plant hormones, such as jasmonic acid (JA), ethylene (ET), salicylic acid (SA), and auxin (IAA) [12,13]. In addition, there are also ABA-independent regulation pathways, such as brassinosteroid (BR), which occur via reactive oxygen species and recover photosynthetic apparatus [14,15]. Auxin also plays a critical role in response to cold stress via polar deployment and intracellular trafficking of auxin carriers [16,17,18].

Lipid and free amino acids contribute greatly to plant cold tolerance and, therefore, are the focus when studying plant cold tolerance [19]. Profiling the transcriptome response to cold stress is the first step to exploring genes and pathways that enable cold survival. Hence, comparative transcriptomic analysis has been carried out in important crops, e.g., *Santalum album* [20], *Nicotiana tabacum* [21], *Bangia fuscopurpurea* [22], and rice [23,24]. Two winter rapeseed (*Brassica raba*) varieties, Longyou-7 (cold-tolerant) and Lenox (cold-sensitive), were compared and subjected to an integrative analysis of morphological, physiological, and transcriptomic changes [25]. Phenylpropanoid biosynthesis, plant hormone signal transduction, ribosome biogenesis, mitogen-activated protein kinase (MAPK) signaling pathway, basal transcription factors, and photosynthesis were suggested to be associated with cold tolerance.

There are already many studies about genetic analysis and QTL mapping of the trait of cold tolerance in cucumbers. Wehner et al. reported the first inheritance study of cucumber chilling resistance in 1992 and suggested a single and dominant gene [26]. An inheritance study of cold-sensitive cucumber GY14 and cold-tolerant cultivar ‘Chipper’ and ‘Little John’, however, suggested maternal inheritance and that chloroplast genomes carry cold-resistant genes [27]. Low-temperature germination ability was controlled by recessive genes in a study of cold-tolerant “PI390953” and cold-sensitive “Gy14” [28]. A dominant single gene, *Ch*, was suggested to control cold tolerance at the seedling stage of PI 246930 [29]. Following that, numerous studies attempted to identify the genetic locus controlling cold tolerance [30,31]. Compared with other divisive QTLs, the loci on chromosome 6 were detected more than once. Inheritance analysis on the cold tolerance of cucumber “0839” indicated a dominant single gene at chromosome 6 by applying 188 polymorphic markers in the F2 population [32]. A more recent study confirmed two major QTLs on chromosome 6, named *qLTT6.1* and *qLTT6.2,* and the major QTL *qLTT6.2* was mapped into a 595-kb interval between markers SSR14859 and SSR21885 [33].

In this study, we evaluated the cold tolerance of 105 accessions to explore gene resources. Plant hormones and transcriptomes were compared between cold-sensitive and cold-tolerant cucumber. These data improved our understanding of the role of plant hormones in cucumber cold tolerance.

## 2. Results

### 2.1. Resistance Evaluation against Low Temperatures in Cucumbers

**Figure 1:**
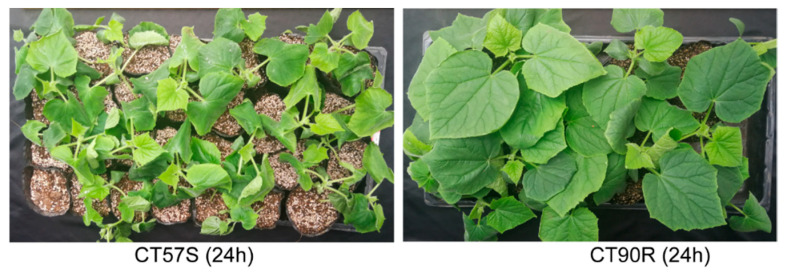
Symptom of chilling injury started to appear at 24 h in cold treatment. **Left**, the appearance of cold-sensitive CT57S. **Right**, the appearance of cold-tolerant CT90R.

Cold tolerance was evaluated every day after exposure to low temperature (4–8 °C), and the index of cold injury on the fifth day had the greatest variation, ranging from 0.03 to 0.98. Therefore, we graded all the accessions according to their tolerance based on the index on the fifth day. A total of 105 germplasm accessions from all over the world were assayed, and finally we identified 10 accessions showing high resistance (Table S1). Cold-resistant CT90 (renamed CT90R hereafter) and cold-sensitive CT57 (renamed CT57S hereafter) were used to study the differences in phytohormones and transcriptome responses to cold treatment. CT90R was so cold-tolerant that there was no visible cold injury after 24 h of treatment (Figure 1).

### 2.2. Plant Hormone Detection

Isopentenyl adenine riboside (IPR), ABA, brassinosteroid (BR), dyhydrozeatin (DZ), indole-3-butyric acid (IBA), inositol phosphate (IP), and jasmonic acid–isoleucine (JA-ILE) were detected in the assayed samples (Table 1). All these phytohormones showed a significant difference between CT90R and CT57S. Interestingly, most of them were higher in CT90R, while only IBA and JA-ILE were lower in CT90R. The content of IPR was 3.26 times greater in CT90R than in CT57S. These results indicate that stable and high-level phytohormones may contribute to cold tolerance.

### 2.3. Distribution of Differentially Expressed Genes (DEGs) on Chromosomes

**Figure 2:**
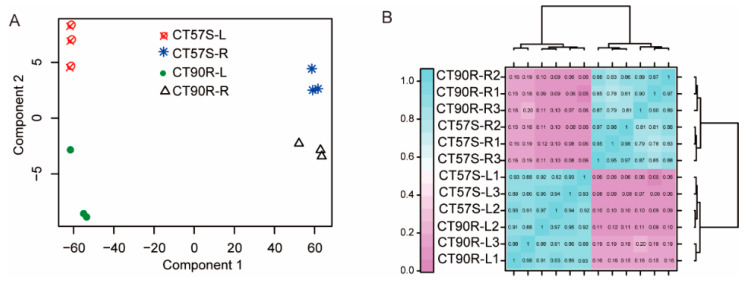
Evaluation of sequencing quality by (**A**) Principal component analysis (PCA) and (**B**) Correlation heat map between samples. CT57S-L, CT57S-leaf; CT57S-L1, the first replicate of CT57S-L; CT57S-R, CT57S-root; CT57S-R1, the first replicate of CT57S-R. The numbers in the heatmap indicate R^2^ value in the correlation analysis.

**Figure 3:**
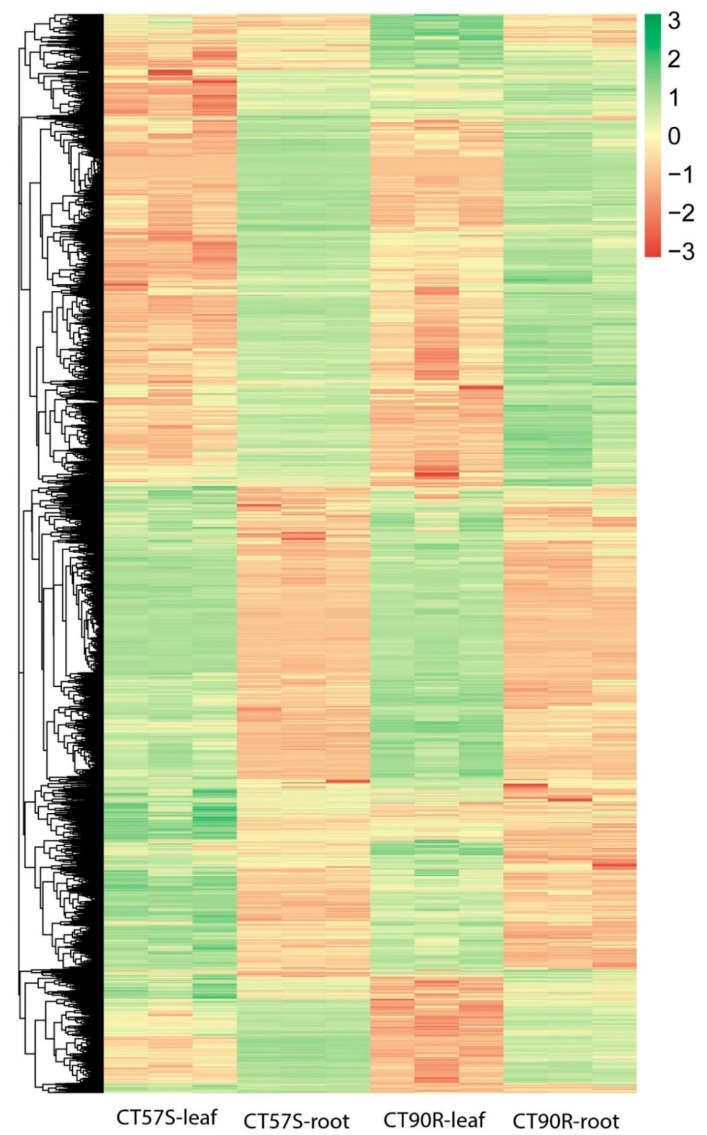
Heat map of DEGs in different samples. DEGs were identified from comparing a pair of CT57S-leaf vs. CT57S-root, CT90R-leaf vs. CT90R-root, CT57S-leaf vs. CT90R-leaf, and CT57S-root vs. CT90R-root. The color represents value of log_10_ (FPKM).

We obtained at least 3 giga base (Gb) of clean data for each RNA-seq, which showed a high alignment rate (96–98%) (Table S2). Triplicate transcriptomes clustered together in principle component analysis (PCA), indicating good RNA-seq quality (Figure 2A). A correlation assessment by Pearson’s Correlation Coefficient indicated a very high R^2^ value (>0.9) between replicates and a high R^2^ value (>0.8) between samples from the same tissue (Figure 2B). There were no abnormal samples. We detected a total of 25230 genes, including 1003 new genes in the transcriptome analysis (Table S3). We detected 9630 DEGs after comparing a pair of CT57S-leaf vs. CT57S-root, CT90R-leaf vs. CT90R-root, CT57S-leaf vs. CT90R-leaf, and CT57S-root vs. CT90R-root, which include 256 new genes. Most of these DEGs show the same expression pattern in the same organs (Figure 3).

**Figure 4:**
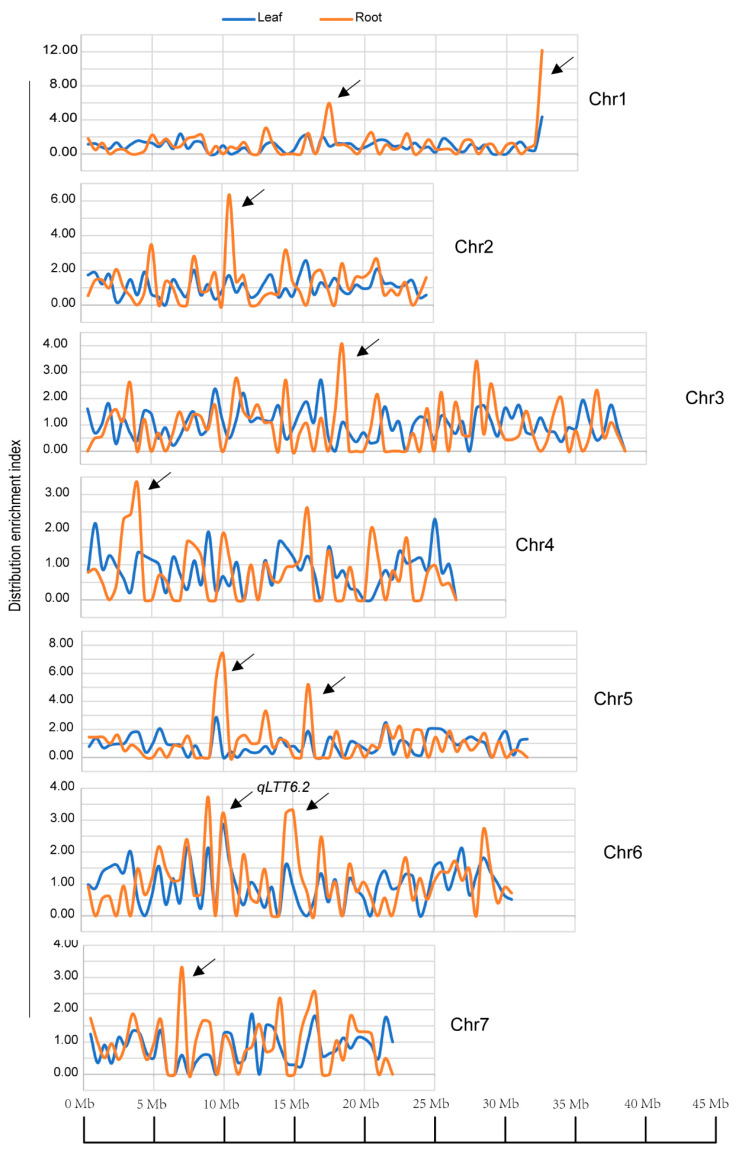
Distribution of DEGs on the seven chromosomes. Distribution enrichment index = (bin DEG number/total DEG number)/(bin gene number/total gene number); bin = 5 kb. The arrows indicate significant enrichments with an index of >3. Blue lines indicate enrichment of DEGs in the leaf (CT57S-leaf vs. CT90R-leaf); orange lines indicate enrichment of DEGs in the root (CT57S-root vs. CT90R-root).

We then inspected the distribution manner on chromosomes of DEGs identified from comparing a pair of CT57S-leaf vs. CT90R-leaf and CT57S-root vs. CT90R-root. There were more DEGs in euchromatin regions rich in protein-coding genes, as anticipated. However, there was still DEG enrichment at particular positions, and ten enrichment regions were identified (Figure 4 and Figure S1). The profile of DEG distribution was different between the leaf and root transcriptomes, although some peaks overlapped. There were much more distribution enrichments in roots than in leaves, and the enrichment index value was much higher. We observed a major enrichment in both leaves and roots around 10 Mb of chromosome 6, which overlapped with *qLTT6. 2*, a major QTL identified by Dong et al. [33]. As many as 22 DEGs were enriched in this region, including CsaV3_6G013600 and CsaV3_6G013960 as auxin-related genes (Table 2). In addition, we identified an interesting DEG in this region which encodes glucan endo-1,3-beta-glucosidase, an enzyme which catalyzes the formation of a cell wall component β-(1,3)-d-glucan. The plant cell wall composition and structure is reported to mediate plant cold tolerance [34].

**Table 2:** Annotation of DEGs enriched at *qLTT6.2*.

Gene ID	Strand	Start	End	Annotation
CsaV3_6G013600	−	9,736,464	9,740,433	Small auxin-up RNA
CsaV3_6G013610	+	9,762,144	9,764,899	Peptidyl-prolyl cis-trans isomerase FKBP5
CsaV3_6G013700	+	9,901,956	9,903,554	NAC domain
CsaV3_6G013720	+	9,919,247	9,921,289	NAC domain
CsaV3_6G013830	+	10,022,293	10,025,625	Glucan endo-1,3-beta-glucosidase
CsaV3_6G013910	−	10,078,532	10,080,455	Aquaporin PIP2-7-like
CsaV3_6G013920	+	10,093,981	10,094,739	Senescence regulator S40
CsaV3_6G013960	−	10,156,022	10,160,149	Auxin response factor
CsaV3_6G014010	−	10,204,862	10,209,042	Exosome complex component MTR3
CsaV3_6G014030	−	10,219,586	10,224,039	Zinc finger
CsaV3_6G014150	+	10,290,872	10,297,455	P-loop NTPase domain
CsaV3_6G014230	−	10,347,201	10,348,923	Heavy metal-associated domain
CsaV3_6G014250	−	10,365,575	10,371,237	Lysine methyltransferase
CsaV3_6G014260	+	10,365,754	10,367,581	Alpha-L-fucosidase 1
CsaV3_6G014270	+	10,371,432	10,374,387	Uncharacterized protein
CsaV3_6G014290	−	10,382,370	10,385,069	Glucuronoxylan glucuronosyltransferase
CsaV3_6G014380	−	10,432,200	10,442,898	Uncharacterized protein
CsaV3_6G014740	−	10,700,897	10,703,058	Histone-lysine N-methyltransferase
CsaV3_6G014750	+	10,709,780	10,710,073	Uncharacterized protein
CsaV3_6G014760	−	10,710,135	10,711,415	Prephenate dehydratase
CsaV3_6G015120	−	10,976,098	10,977,090	Uncharacterized protein
CsaV3_6G015130	−	10,978,344	10,982,661	Protein trichome birefringence-like 11

### 2.4. Differential Expression between Leaves and Roots

**Figure 5:**
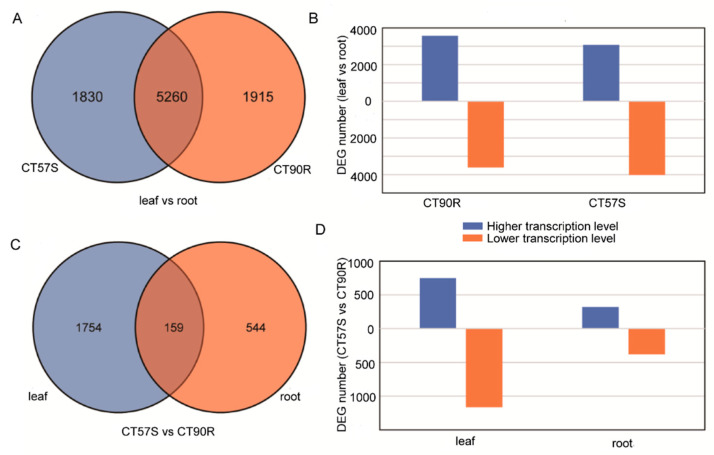
DEGs identified in different comparing pairs. (**A**,**B**) are statistical data of DEGs in comparing a pair of leaf vs. root. (**C**,**D**) are statistical data of DEGs in comparing a pair of CT57S vs. CT90R.

A total of 16834 genes were detected in leaves, while 17,148 were detected in cucumber roots. Among them, 1206 genes were leaf-specific and 1520 genes were root-specific. In comparison to leaf vs. root, there were as many as 9005 DEGs, among which 5260 DEGs were shared by the two cucumbers, indicating a big difference between the leaf and root transcriptome (Figure 5A). The number of upregulated DEGs and downregulated DEGs was nearly the same (Figure 5B). Significantly enriched GO terms were identified by corrected *p*-Value, and the top five items in GO enrichment were: plastid organization, chloroplast organization, plastid membrane organization, thylakoid membrane organization, and photosynthesis (Table 3).

### 2.5. Differential Expression in Leaves between Cold-Tolerant and Cold-Sensitive Cucumber

**Figure 6:**
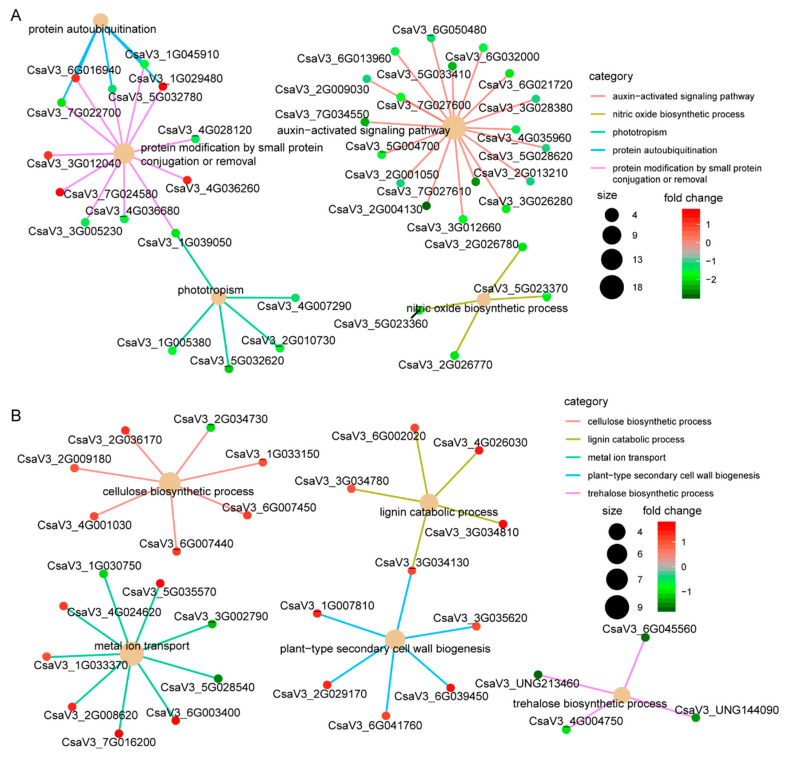
Cnetplot diagram showing the most significantly enriched 5 items in GO analysis (a biological process). (**A**) Comparing a pair of CT57S vs. CT90R in leaves. (**B**) Comparing a pair of CT57S vs. CT90R in roots.

We identified a total of 1913 DEGs between CT90R and CT57S in leaves. These DEGs were assigned to GO in terms of growth, immune system processes, signaling, response to stimuli, etc. (Figure S2A). In the enrichment analysis, the top five GO terms were the auxin-activated signaling pathway, the nitric oxide biosynthetic process, phototropism, protein autoubiquitination, and protein modification by small protein conjugation or removal (Figure 6A). For the auxin-activated signaling pathway, there were as many as 19 DEGs. A network was found between protein autoubiquitination, protein modification, and phototropism.

**Figure 7:**
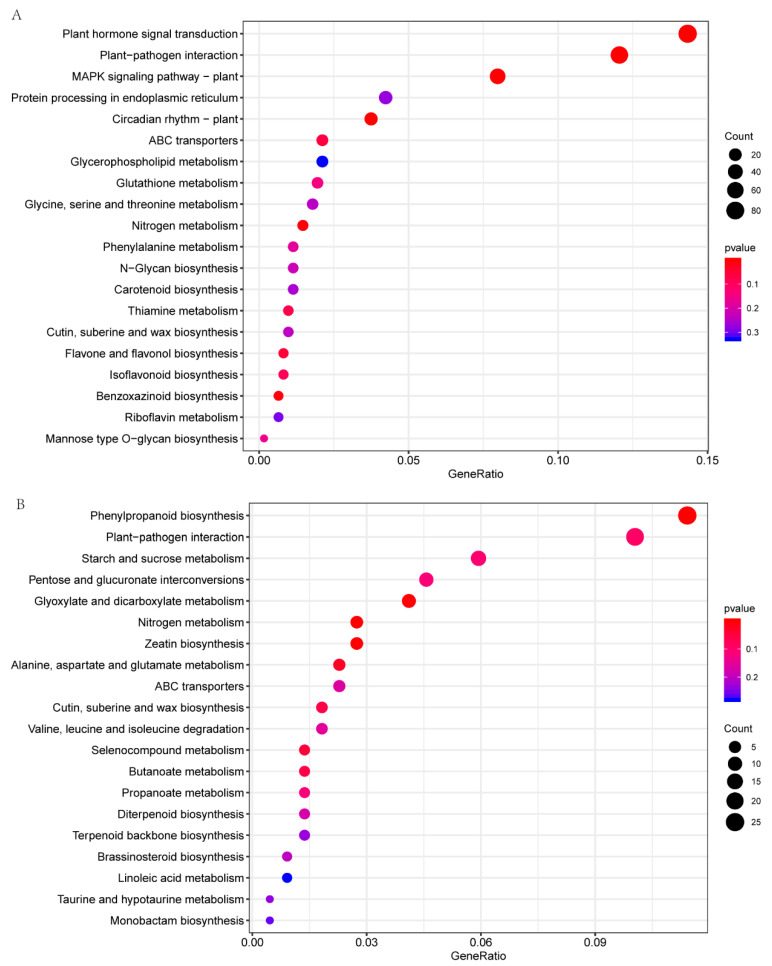
Dot plot diagram showing the most significantly enriched 20 items in KEGG analysis. (**A**) Comparing a pair of CT57S vs. CT90R in leaves. (**B**) Comparing a pair of CT57S vs. CT90R in roots.

**Figure 8:**
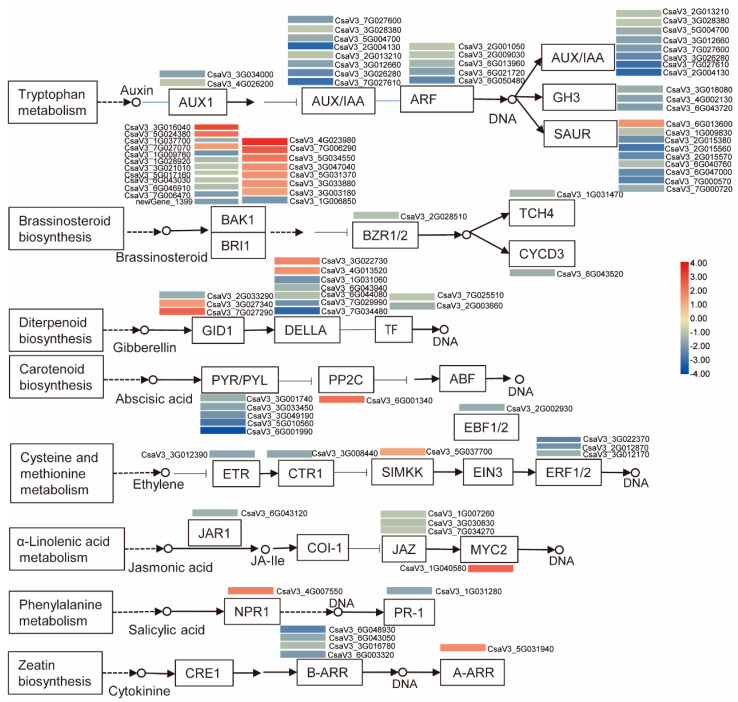
Processes of plant hormone signal transduction were significantly different between CT57S and CT90R in leaves. The process map was modified based on map ko04075.

Similarly, KEGG enrichment analysis also indicates the important role of plant hormones. The most significant enrichment is in plant hormone signal transduction (ko04075), followed by plant–pathogen interaction (ko04626) and MAPK signaling pathway–plant (ko04016) (Figure 7A). There were 97 DEGs enriched in plant hormone signal transduction, and we further analyzed these pathway-related DEGs (Figure 8). The processing of auxin had the biggest number of DEGs, followed by BR and GA. As demonstrated in signal transduction of abscisic acid and ethylene, downregulation of negative regulators resulted in the upregulation of downstream targeted genes, implying high reliability of this pathway analysis. Most of the DEGs were down-regulated in CT90R, especially in auxin signal transduction and brassinosteroid signal transduction. The pathway of auxin signal transduction was clearly and totally suppressed in CT90R in the cold treatment.

### 2.6. Differentially Expression in Roots between Cold-Tolerant and Cold-Sensitive Cucumber

We identified a total of 703 DEGs between CT90R and CT57S in roots. These DEGs were annotated with GO terms of detoxification, growth, the system process, localization, and the rhythmic process (Figure S2B). The cellulose biosynthetic process, the lignin catabolic process, plant-type secondary cell wall biogenesis, metal ion transport and the trehalose biosynthetic process were the top five GO terms with significant enrichment (Figure 6B). The most significant enrichment in KEGG analysis was observed in phenylpropanoid biosynthesis (ko00940), followed by plant–pathogen interaction, starch, and sucrose metabolism (Figure 7B). These above results indicate a distinct mechanism of cold resistance in roots, which are associated with lignin biosynthesis and cell wall construction.

**Figure 9:**
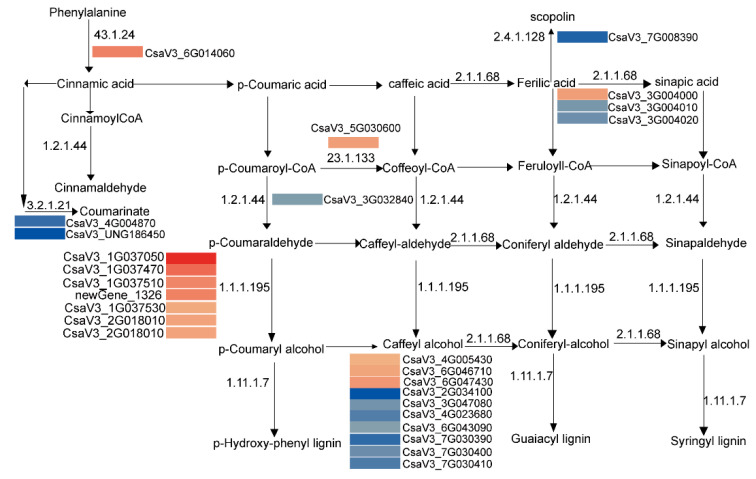
Process of phenylpropanoid biosynthesis was significantly different between CT57S and CT90R in roots. The process map was modified based on the map ko00940.

As enrichment of phenylpropanoid biosynthesis is notably significant in roots, we further analyzed this pathway (Figure 9). Seven genes that encode cinnamyl-alcohol dehydrogenase (CAD) were upregulated in CT90R; however, seven genes that catalyze the downstream step of lignin biosynthesis were downregulated. These results together imply a higher content of monolignols in CT90R, which include *p*-coumaryl alcohol, caffeyl alcohol, coniferyl alcohol, and sinapyl alcohol.

## 3. Discussion

Five indexes, including the chilling injury index, the seedlings surviving rate, the number of leaves, the plant height, and the chlorophyll content, were chosen to explore the best methodology of cold tolerance identification [35]. There was a significantly negative correlation between the chilling injury index and the other four indexes; the chilling injury index contributed to the first component in PCA analysis [35]. By directly using the chilling injury index, we identified 10 germplasm accessions with high cold tolerance from 105 accessions, 20 of which were from Sichuan province, China. There have been many reports about cold tolerance identification in cucumbers, but the number of germplasms is limited in most studies. For example, Ali et al. compared cold tolerance of 12 commercial cucumber varieties in Korea [36]. In general, cold-tolerant cucumbers are rare in germplasm and the proportion of cold-tolerant cucumber is lower than 10% [35,37]. Further research is required in order to explore and utilize more genetic resources. Alternatively, genetic engineering approaches, such as gene editing, should be adopted in the future. Anyhow, clear regulatory and genetic mechanism of cold tolerance in cucumbers are prerequisites for these applications.

In the current study, 17 phytohormones were studied by using the LC-MS/MS method. Seven hormones (IPR, ABA, BR, DZ, IBA, IP, and JA-ILE) were significantly different between CT90R and CT57S. CT90R had higher phytohormone levels of ABA, IP and IPR (a cytokinin), BR, and DZ (a zeatin) than CT57S. Similarly, increased levels of these plant hormones were observed during cold acclimation in the cold-tolerant *Lolium perenne* [38]. ABA can activate the cold acclimation process, and the exogenous application of ABA is known to induce cold tolerance. Furthermore, BR, cytokinin, and zeatin are also known to enhance cold tolerance [12,13]. In addition to plant hormone contents, eight pathways of hormone signal transduction were significantly different between CT90R and CT57S, particularly for auxin, ABA, BR, and GA (Figure 8). In maize, these molecules have been found to induce cold acclimation, resulting in a higher tolerance to low temperatures [39,40]. The fluctuation of plant hormone signal transduction during cold treatment seems a common phenomenon, as found in many other plants [41,42,43,44].

Our analysis suggested a wide distribution of DEGs across all seven chromosomes. However, we observed a major distribution enrichment in both leaves and roots at the position of 10 Mb of chromosome 6, which overlapped with the previously reported major QTL named *qLTT6.2*. This locus was positioned between markers SSR14859 and SSR21885 with an interval of 595 kb, and a closely linked marker SSR07248 was developed [32,33]. In this region, we found two auxin-related genes (CsaV3_6G013600 and CsaV3_6G013960) that encode small auxin-up RNA and an auxin response factor (ARF), respectively. Intracellular regulation responding to environmental change is sometimes reported to occur in a manner of clustered genes, which may result from chromatin modification.

The mechanism of cold tolerance in roots might be different from leaves, since DEGs in roots and leaves are different when compared between CT90R and CT57S. The most significantly enriched pathway in leaves was plant hormone signal transduction (ko04075), while phenylpropanoid biosynthesis (ko00940) was enriched in the root, and there were as many as 26 DEGs in this pathway. Upregulation of phenylpropanoid biosynthesis was reported in cucumbers when pre-storage cold acclimation was applied to enhance plant cold tolerance [45]. In maize, 14 genes of the phenylpropanoid biosynthesis pathway were significantly upregulated in tolerant lines [46]. Our study further implies that CT90R may accumulate more monolignols, including *p*-coumaryl alcohol, caffeyl alcohol, coniferyl alcohol, and sinapyl alcohol, by upregulating CAD (1.1.1.195) and downregulating peroxidase (1.11.1.7). The role of CAD in plant cold acclimation and cold tolerance was stressed in a recent important review paper, and enhanced lignin synthesis was suggested to strengthen cell wall [47]. Greatly induced or upregulated CAD activity was observed in cold-tolerant *Miscanthus* spp. [48], *Arabidopsis* [49], and sweet potato [50]. The three monolignols (*p*-coumaryl, coniferyl, and sinapyl alcohols) were synthesized in the cytosol [51], and were then transported from the cytosol to different locations in the cell wall, resulting in resistance to diverse stresses in plants. The overexpression of IbCAD1, which catalyzes the formation of monolignols, could enhance cold storage of sweet potato. In addition, high monolignol levels could serve as substrates for peroxidase activity [52]. Metabolome analysis of roots needs to be conducted to clarify the possible more accumulation of monolignols in tolerance cucumbers.

## 4. Plant Materials and Methods

### 4.1. Plant Materials and Field Investigation

We investigated the cold tolerance of 105 cucumber accessions by exposing them in the winter (Table S1). These germplasms were collected from all over the world. All the seedlings were grown in pots that were placed in a small plastic tunnel in the green house in Chongzhou, Sichuan Province, China. A double greenhouse enabled normal growth in early winter. For each accession, there were 25 seedlings. The seeds were sown on January 1st and the seedlings were kept in a double greenhouse until 28th January, when the 3rd leaves unfolded (Figure S3). Before complete exposure to natural cold stress, all the seedlings were acclimated for 9 days by removing the inner plastic tunnel. The index of cold injuries was recorded according to their appearance on the 5th day when the big green house was open.

Cold-tolerant cucumber CT90R and cold-sensitive cucumber CT57S were used for phytohormone detection and RNA-seq. Seedlings were grown in plug pots and placed in incubators with an environmental condition of 25 °C, light (12 h/12 h, day/night), and RH of 80%. When the fourth true leaf unfolded, the seedlings were acclimated to 16 °C for one day, and finally treated at a low temperature of 6 °C. The second unfolded leaf and the entire root were separately collected at 24 h during the cold treatment from 15 seedlings. Leaves or roots from five seedlings were pooled as one sample, and there were triplicates. Each sample was rapidly frozen by immersing the sample tube in liquid nitrogen and stored at −80 °C. The same leaf samples were used in phytohormone detection and RNA-seq.

### 4.2. Detection of Phytohormone

Phytohormones in cucumber leaves were detected by the LC-MS/MS method. Leaves were homogenized with liquid nitrogen. About 100 mg of tissue powder was suspended in 200 L of pre-cooled water and then used to make a methanol–acetonitrile (2:2, *v*/*v*) extraction. After 60 min of extraction in an ice-bath using ultrasonic, the peptide precipitate was incubated at −20 °C and then centrifuged at 14,000× *g* for 20 min. The supernatant was dried by a rotary evaporator and the residue was resolved in 200 μL of acetonitrile. The precipitate was discarded by centrifuging at 20,000 g for 10 min.

The phytohormone extraction was separated by ultrahigh-pressure liquid chromatography (Nexera X2 LC-30AD, Shimadzu, Japan). Mobile phase A was 10 mM ammonium acetate (pH8.0), while mobile phase B was 100% acetonitrile. A five-microliter solution was ejected at 4 °C with a column temperature of 40 °C; the mobile phase speed was 300 μL/min. The gradient ration of mobile phase B was: 0–0.5 min, 5%; 0.5–6.5 min, increased to 65%; 6.5–8 min, increased to 98%; 8–9.9 min, kept at 98%; 9.9–10 min, decreased to 5%; and 10–12 min, kept at 5%. The mass spectrometry was 5500QTRAQ (AB SCIEX) and the conditions were: source temperature—550 °C, ion source gas1 (GAS1)—40, ion source gas2 (GAS2)—50, curtain gas (CUR)—35, positive ion spray voltage floating (ISVF)—5500V, negative ion spray voltage floating (ISVF)—4500 V, and multiple reaction monitoring (MRM). A standard mixture, including as many as 17 phytohormones, was used in LC-MS/MS detection to make a standard curve (Table S4). All the phytohormones were well separated by the presented method. Results of quality control indicated reliable and reproducible detection (Table S5).

### 4.3. RNA-Seq

Total RNA was extracted using the Trizol method, as previously described [53]. In total, 1 μg of RNA per sample was used as input material for the RNA sample preparations. The purity, concentration, and integrity of the RNA sample were examined by NanoDrop, Qubit 2.0, Agilent 2100 (Agilent Technologies, de novo Santa Clara, CA, USA). Qualified RNA was processed for library construction and illumine sequencing by Biomarker (Beijing, China).

The PHRED quality score was calculated to evaluate the probability of an incorrect base [54]. Clean data were obtained after trimming adapter contaminations and removing low-quality-score nucleotides. Clean reads were mapped to the Chinese Long *Cucumis sativus* genome (version 3.0) (http://cucurbitgenomics.org/ftp/genome/cucumber/Chinese_long/v3/ (accessed on 1 January 2021)) by using an alignment tool with high-efficiency HISAT2 [55]. String tie with an algorithm based on optimality theory was applied to assemble the mapped reads [56]. Novel genes were annotated by DIAMOND against databases including NR, SWISS-PROT, COG, KOG, and KEGG [57]. Fragments per kilobase of transcript per million fragments mapped (FPKM) were applied to measure the expression level of a gene or transcript by StringTie using maximum flow algorithm [56]. Pearson correlation coefficient R was applied in this project to evaluate the reproducibility of biological replicates [58]. Differential expression analysis was processed by DESeq2, and the criteria for differentially expressed genes were set as fold change (FC) ≥ 2 and FDR < 0.01 [59]. Functional annotation and enrichment analysis were conducted using a database of gene ontology (GO) and the Kyoto Encyclopedia of Genes and Genomes (KEGG).

## 5. Conclusions

From 105 germplasm accessions, we screened out 10 accessions with high resistance to cold stress. A higher content of plant hormones was observed in the leaves of CT90R, which was accompanied by a lower expression of plant hormone transduction revealed by transcriptome comparison. As many as 22 DEGs, including 2 auxin signaling-related genes, were significantly enriched at *qLTT6.2*, i.e., a major QTL controlling cucumber cold tolerance. In roots, CT90R had higher expression levels of upstream genes of 4-hydroxycinnamyl alcohols and lower expression levels of downstream genes, indicating a relative higher content of 4-hydroxycinnamyl alcohols.

## Figures and Tables

**Table 1:** Plant hormone identified in cucumber leaves in cold treatment.

Plant Hormone ^1^	RT (min) ^2^	Content (ng/g)		FC ^3^	*p*-Value ^4^
		CT57S	CT90R		
IPR	5.35	0.88	0.27	3.26	0.000
ABA	4.1	28.22	23.06	1.22	0.015
BR	7.93	17.30	5.41	3.20	0.010
DZ	4.02	1.02	0.51	2.00	0.023
IBA	4.4	5.28	10.35	0.51	0.001
IP	5.4	1.58	1.26	1.25	0.004
JA-ILE	5.25	3.05	8.50	0.36	0.003

^1^ IPR, isopentenyladenine riboside; ABA, abscisic acid; BR, brassinolide; DZ, dihydrozeatin; IBA, 3-indolebutyric acid; IP, N6-isopentenyladenine; JA-ILE, jasmonic acid–isoleucine. ^2^ RT, retention time. ^3^ FC, fold change in the peak area = CT90R/CT57S. ^4^ *p*-value of two-sample *t*-test.

**Table 3:** Top 10 items in GO enrichment analysis of DEGs identified between leaves and roots.

GO ID	GO Term	Genome Frequency ^1^	CT57S		CT90R	
			DEG Num	Cluster Frequency	DEG Num	Cluster Frequency
GO:0009657	plastid organization	0.90%	172	3.00%	172	2.90%
GO:0009658	chloroplast organization	0.60%	107	1.80%	108	1.80%
GO:0009668	plastid membrane organization	0.50%	98	1.70%	97	1.60%
GO:0010027	thylakoid membrane organization	0.50%	98	1.70%	97	1.60%
GO:0015979	photosynthesis	0.90%	187	3.20%	189	3.20%
GO:0019682	glyceraldehyde-3-phosphate metabolic process	0.90%	150	2.60%	145	2.40%
GO:0019684	photosynthesis, light reaction	0.60%	137	2.40%	138	2.30%
GO:0044699	single-organism process	28.70%	2092	36%	2173	36.40%
GO:0044710	single-organism metabolic process	20.20%	1586	27.30%	1637	27.40%
GO:0055114	oxidation–reduction process	7.70%	694	11.90%	758	12.70%

^1^ Genome frequency, the number of genes annotated to a GO term in the entire background set.

## Data Availability

The data presented in this study are openly available in NCBI: BioProject ID, PRJNA817708.

## Supplementary Materials

The following supporting information can be downloaded at: https://www.mdpi.com/article/10.3390/plants11070961/s1. Figure S1: Distribution of DEGs on chromosomes. Figure S2: GO classification of DEGs. Figure S3: Temperature decreased during the cold treatment in winter. Table S1: Cold tolerance of cucumber inbred lines. Table S2: Raw data of RNA-seq. Table S3: New genes identified in transcriptome assemblies. Table S4: The standard curve in plant hormone detection. Table S5: Quantity control in plant hormone detection.
