# Supplementary material for: Higher Phytohormone Contents and Weaker Phytohormone Signal Transduction Were Observed in Cold-Tolerant Cucumber
Source: Plants (Basel). 2022 Apr 1;11(7):961. doi: 10.3390/plants11070961 (PMC9003209; doi:10.3390/plants11070961)
Supplement: Supplementary file 1 [file plants-11-00961-s001.zip › Supplementary Materials Figures.pdf]

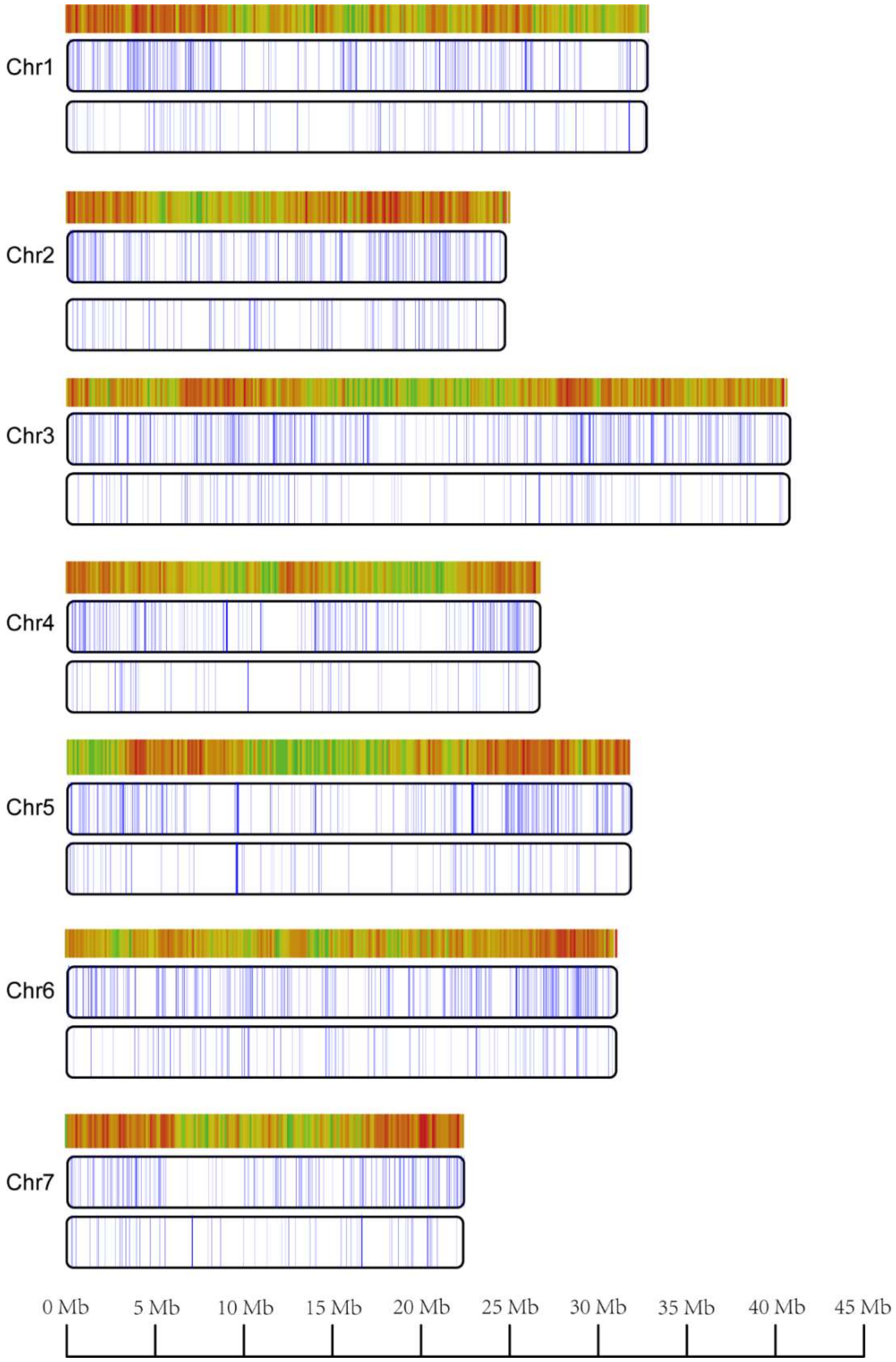

Figure S1. Distribution of DEGs on the seven chromosomes. The three columns from top to bottom for each chromosome indicate genomic gene distribution, DEG (CT57S v.s. CT90R) in leaves, and DEG (CT57S v.s. CT90R) in roots.

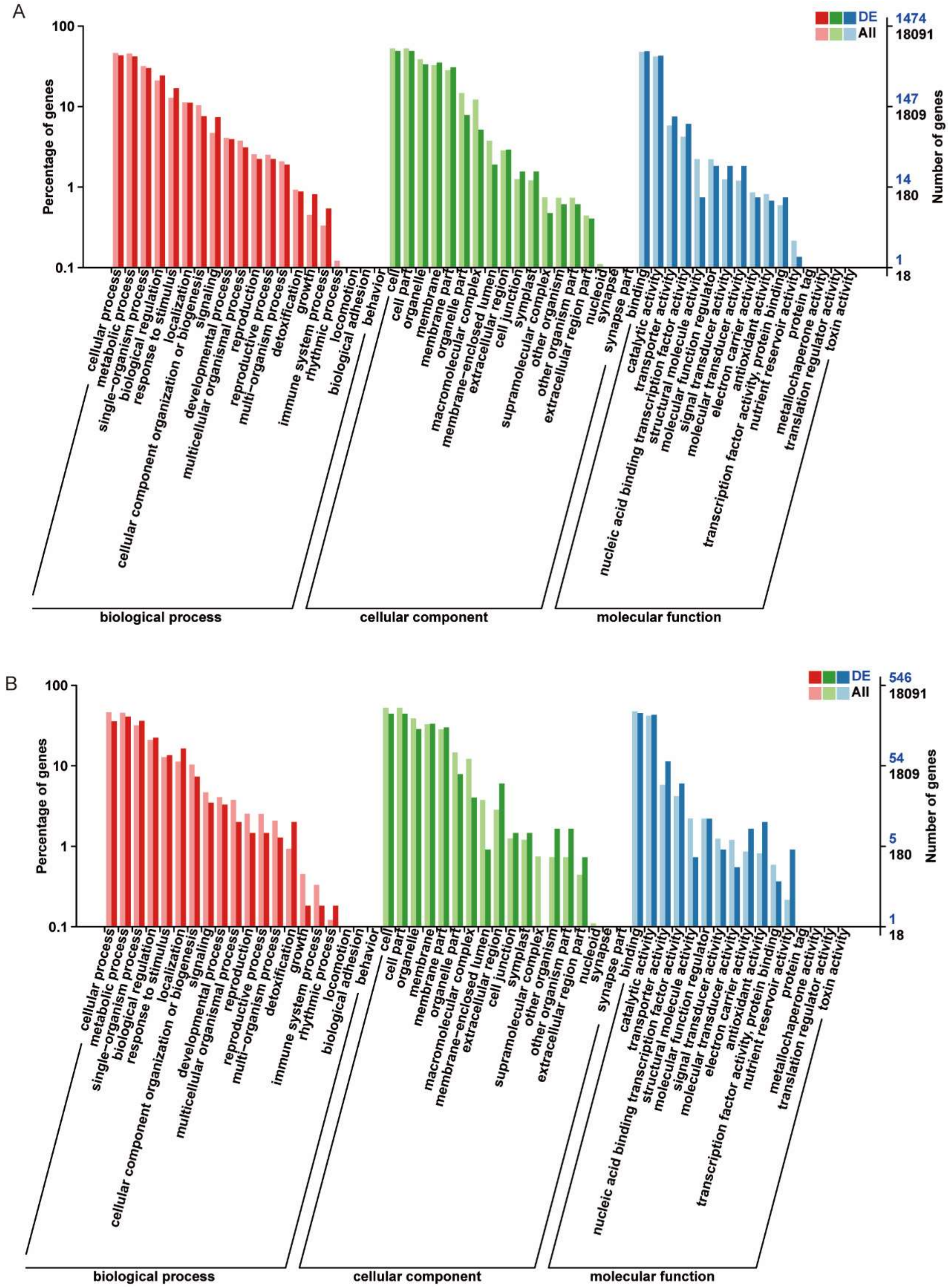

Figure S2. GO classification of DEGs. (A) Comparing pair of CT57S v.s. CT90R in leaves. (B) Comparing pair of CT57S v.s. CT90R in roots.

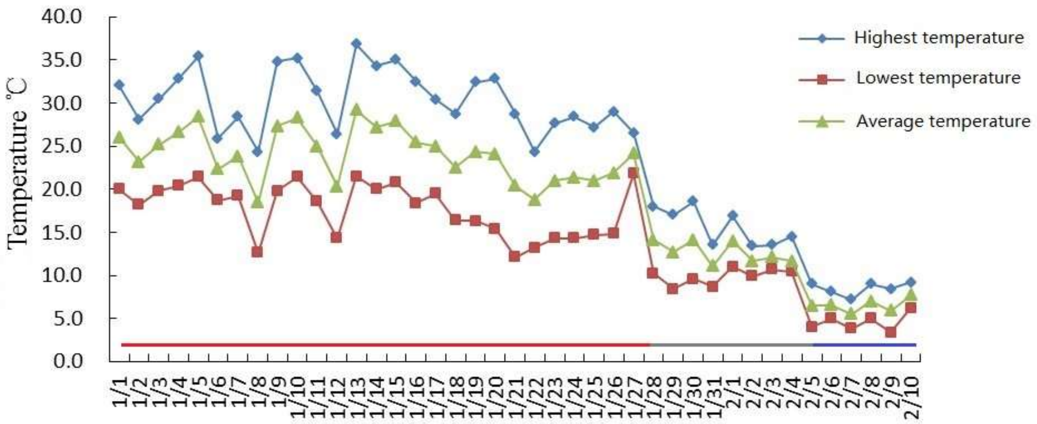

Figure S3. Temperature decreased during the cold treatment in winter.
